# National Influenza Surveillance in the Philippines from 2006 to 2012: seasonality and circulating strains

**DOI:** 10.1186/s12879-016-2087-9

**Published:** 2016-12-19

**Authors:** Marilla G. Lucero, Marianette T. Inobaya, Leilani T. Nillos, Alvin G. Tan, Vina Lea F. Arguelles, Christine Joy C. Dureza, Edelwisa S. Mercado, Analisa N. Bautista, Veronica L. Tallo, Agnes V. Barrientos, Tomas Rodriguez, Remigio M. Olveda

**Affiliations:** 1 Department of Health, Research Institute for Tropical Medicine, Filinvest Corporate City, Alabang, Muntinlupa City, Philippines; 2 Centers for Disease Control and Prevention, Atlanta, GA USA

**Keywords:** Influenza seasonality, Average epidemic curve, Seasonal threshold, Alert threshold, Circulating influenza strains, Influenza vaccine strains

## Abstract

**Background:**

The results of routine influenza surveillance in 13 regions in the Philippines from 2006 to 2012 are presented, describing the annual seasonal epidemics of confirmed influenza virus infection, seasonal and alert thresholds, epidemic curve, and circulating influenza strains.

**Methods:**

Retrospective analysis of Philippine influenza surveillance data from 2006 to 2012 was conducted to determine seasonality with the use of weekly influenza positivity rates and calculating epidemic curves and seasonal and alert thresholds using the World Health Organization (WHO) global epidemiological surveillance standards for influenza.

**Results:**

Increased weekly influenza positive rates were observed from June to November, coinciding with the rainy season and school opening. Two or more peaks of influenza activity were observed with different dominant influenza types associated with each peak. A-H1N1, A-H3N2, and two types of B viruses circulated during the influenza season in varying proportions every year. Increased influenza activity for 2012 occurred 8 weeks late in week 29, rather than the expected week of rise of cases in week 21 as depicted in the established average epidemic curve and seasonal threshold. The intensity was severe going above the alert threshold but of short duration. Southern Hemisphere vaccine strains matched circulating influenza virus for more surveillance years than Northern Hemisphere vaccine strains.

**Conclusions:**

Influenza seasonality in the Philippines is from June to November. The ideal time to administer Southern Hemisphere influenza vaccine should be from April to May. With two lineages of influenza B circulating annually, quadrivalent vaccine might have more impact on influenza control than trivalent vaccine. Establishment of thresholds and average epidemic curve provide a tool for policy-makers to assess the intensity or severity of the current influenza epidemic even early in its course, to help plan more precisely resources necessary to control the outbreak. Influenza surveillance activities should be continued in the Philippines and funding for such activities should already be incorporated into the Philippine health budget.

**Electronic supplementary material:**

The online version of this article (doi:10.1186/s12879-016-2087-9) contains supplementary material, which is available to authorized users.

## Background

The World Health Organization recommends that seasonal and alert thresholds and average epidemic curves be established in countries as tools for early detection of influenza outbreaks to help control annual influenza epidemics [1, 2]. Seasonal influenza epidemics occur all over the world and cause substantial economic burden through health care costs and absenteeism [3]. Although surveillance is now routinely conducted in many countries providing seasonality data and recommendations for timely vaccination [4, 5], not all countries have established influenza thresholds or epidemic curves. Developed countries like the United States (US), United Kingdom (UK), most European countries, Australia, and New Zealand [6–10] are already using thresholds for influenza surveillance calculated through various methods [11, 12]. In contrast, few countries in Asia have established their own thresholds and epidemic curves to determine if an influenza outbreak has started while concurrently monitoring influenza activity [2]. The purpose of this article is to report the results of Philippine National Influenza Surveillance from 2006 to 2012, particularly on the establishment of seasonal and alert thresholds, and average epidemic curve according to methods in the WHO manual [1], define the seasonality of influenza, and describe the circulating influenza strains.

## Methods

### Study design

We retrospectively analyzed weekly trends in influenza virus activity using Philippine National Influenza Surveillance data from 2006 to 2012.

### Surveillance site

The Philippines is located within the latitude and longitude of 13° 00′ N, 122° 00′ E [13]. The Philippines includes 300,000 km^2^ of total land area and is divided into three island groups: Luzon, Visayas, and Mindanao. It is administratively divided into 17 regions. It has a tropical climate with the rainy season from June to November and the dry season from December to May [14].

### Establishment of influenza sentinel sites

**Fig. 1:**
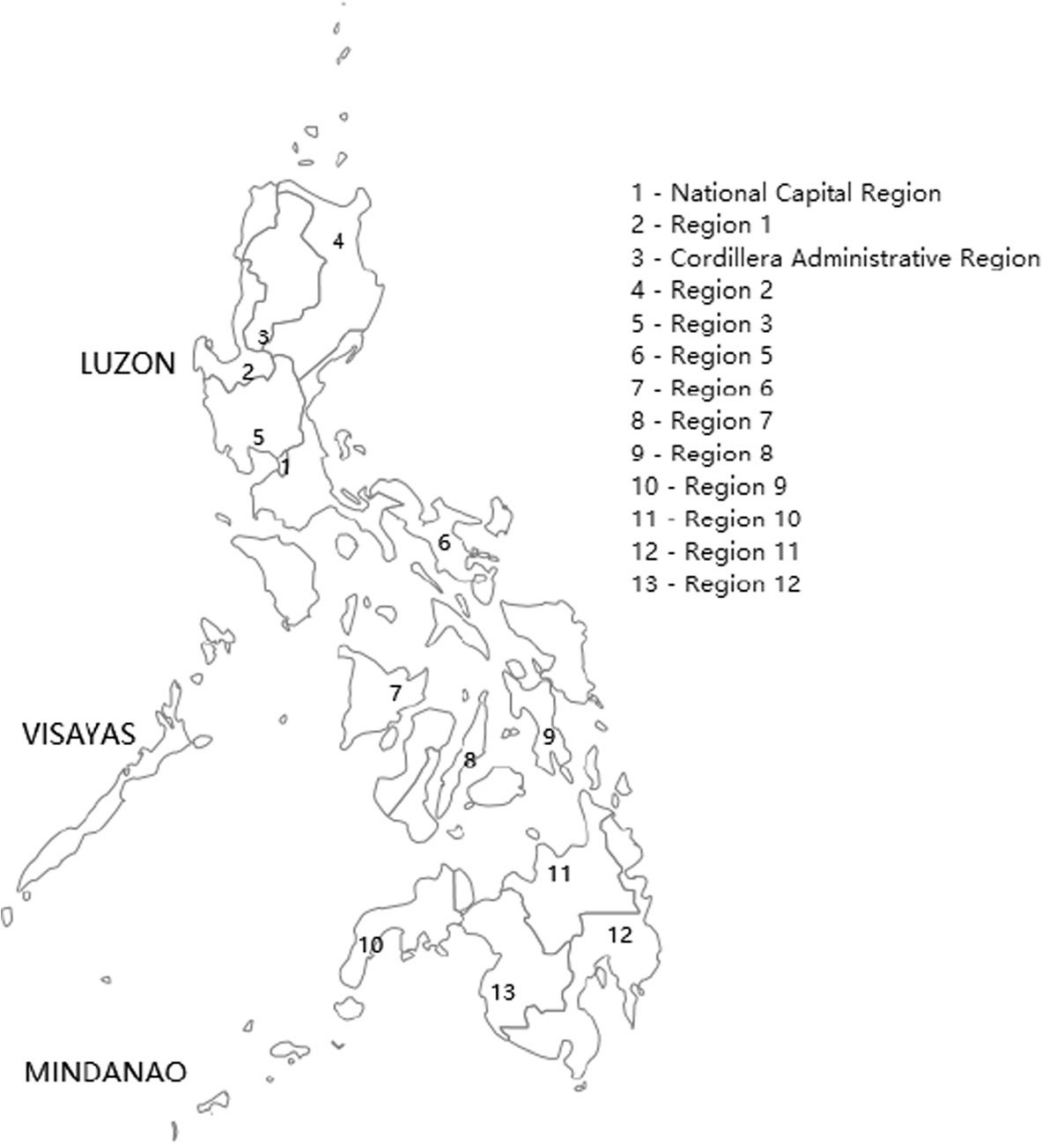
Map of the Philippines showing the sentinel sites (areas with numbers) participating in the national influenza surveillance network. (The map was created using Epi Info Map of Epi Info 7)

In 2004, the Research Institute for Tropical Medicine (RITM) became a National Influenza Center (NIC) and received a grant from the Centers for Disease Control and Prevention (CDC) in the United States (US), to establish a National Influenza Surveillance Network. Surveillance activities started in ten sentinel sites in five regions in June 2005. From 2006 to October 2008, the sentinel sites had increased to 36 distributed in 13 out of 17 regions in the country. There were 18 health centers and 18 Outpatient Departments (OPD) of tertiary hospitals. Eight of the health centers were located in the National Capital Region (NCR) (Fig. 1). In November 2008, a CDC-funded influenza Burden of Disease study (BOD) [15] was set up with 16 health centers in the Cordillera Autonomous Region (CAR) (Fig. 1). The NIC asked the investigators to contribute influenza virus infection data to the national influenza surveillance. There were now 52 sentinel sites by 2009. Sentinel sites were chosen based on the high number of clinic consultations among the health centers in the city, a high population density, and the region being a migratory pathway of birds mixing with local population of fowls and poultry.

### Data collection

#### Clinical and epidemiologic data on influenza-like-illness

Clinical and epidemiologic data were collected in health centers and outpatient departments or admissions in hospitals designated as influenza surveillance sites, from patients with influenza-like-illness (ILI). The health center is the first line of primary health care in the Philippines, and is located in the community it is serving with a typical population of 15,000 to 250,000. One physician, one nurse, one midwife, one medical technologist and one sanitary inspector usually man the health center. It is responsible for the delivery of the health programs of the Department of Health. These health services include but are not limited to morbidity consultations and management, expanded program of immunization, prenatal care, family planning services and such disease control programs like tuberculosis, diarrhea, and acute respiratory infections. As the first line of primary health care, sick individuals who patronize the services at the health centers are either mildly to severely sick. Severely ill patients consulting the center are immediately referred to the hospitals.

Data collected were written into case report forms and included signs and symptoms of cough, fever, sore throat, rhinitis or colds, and history of travel and treatment received for illness before consulting the health center. ILI was defined as any patient with history of fever of sudden onset or measured temperature of >37.7 °C and cough or sore throat [16]. In children ≤3 years of age, ILI was defined only as fever and colds. “Colds” in these children was synonymous with runny nose. Sentinel sites were visited twice a week (Monday and Tuesday) to collect nasopharyngeal (NP) or oropharyngeal (OP) swabs from patients with ILI. Only clinical information and epidemiologic data were collected from Wednesday to Friday.

### Specimen collection and transport of specimens

A standard operating procedure was created for the collection and transport of appropriate clinical specimens (NP or OP swabs) from patients with ILI throughout each year and especially during the influenza season or suspected outbreaks. Influenza surveillance officers (ISO) employed by the project or trained personnel like midwives or nurses in the sentinel sites collected the NP/OP swabs. Swabs were transferred to virus transport medium (VTM) and stored in refrigerators before transporting to RITM in cold boxes with ice or frozen gel packs through private couriers or by ISO within a day or two of specimen collection.

### Identification of influenza virus

For the ILI sentinel surveillance clinical specimens (NP or OP swabs), we used viral culture to detect influenza virus and other respiratory viruses (Parainfluenza 1,2,3, Respiratory Syncytial Virus (RSV), Herpes Simplex Virus (HSV), adenovirus, enterovirus and rhinovirus) using standard procedures recommended by the WHO Global Influenza Surveillance Network (WHO-GISN) [17]. From viral culture, direct Immunofluorescence (IF) test of the Imagen kit was used to identify influenza virus types A and B. We performed Hemagglutination Inhibition (HAI) test using the WHO Influenza Reagent kit provided by the WHO Collaborating Centre for Reference and Research on Influenza (WHO-CCRI), Melbourne, Australia for further influenza virus strain characterization. For the confirmation of Parainfluenza 1, 2, 3, RSV, HSV and Adenovirus in viral culture, we used Immunofluorescence test while Acid stability testing was used to identify enterovirus and rhinovirus.

The influenza BOD study used real-time reverse-transcriptase polymerase chain reaction (RT-PCR) to detect Influenza A from clinical specimens (NP or OP swabs) using the US CDC method or the Applied Biosystems Pandemic H1N1/09 Assay Set v. 2.0 (Life Technologies, CA, USA) [18]. Further subtyping was done to identify seasonal H1 and H3 subtypes using the method of the Centre for Health Protection, Department of Health, Hong Kong SAR [19] and Pandemic H1N1 2009 subtype [18] for Influenza A positive samples. Conventional RT-PCR methodology was used to detect Influenza B and RSV [20].

### Data management and statistical analysis

The National Influenza Surveillance database containing information from CRFs that were initially encoded into EPI-INFO software version 6.04 (public domain statistical software from CDC) was used to obtain data on weekly counts of ILI cases, ILI cases with and without specimens, and those that were positive for influenza and other viruses. Analysis was conducted using MS-Excel, STATA 10 (STATA Corporation, College Station, TX, USA), and R 2.15.0 software [21]. We calculated seasonal and alert thresholds, and epidemic curves using both counts (number of influenza positive cases) and proportions (percent positivity rates). We chose to present the results according to percent positivity rates per week.

### Determination of influenza season and seasonal threshold

Seasonal threshold was defined as the level of influenza activity that signalled the start and end of the annual influenza season [1]. We defined influenza season as the period in which weekly influenza positive rates were above the average influenza positivity rate for a particular surveillance year for at least three consecutive weeks. Weekly influenza positivity rate was the per cent of ILI with specimens per week, which were positive for influenza virus. We noted the onset, end, and duration of the influenza season for each surveillance year. The onset of influenza season was the week in which positivity rate was above the weekly average positivity rate for that year and which continued for three consecutive weeks. The end of the influenza season was the first week in which the positivity rate was below the average weekly positivity rate for that year and which continued for three consecutive weeks. Once the onset and end of the influenza season was known, weekly data from this period were deleted creating a pre- and post-influenza period. This was done for surveillance years 2006, 2007, 2008, 2010, and 2011 and the average weekly influenza positivity rate for all these years was determined and considered as the seasonal threshold. We excluded 2009 because a pandemic year would not reflect findings for a regular influenza season.

### Determination of average epidemic curve and alert threshold

We used the weekly influenza positivity rates for the calculation of average epidemic curve and alert threshold utilizing the 2006, 2007, 2008, 2010 and 2011 data. The average epidemic curve was the usual level of influenza activity in and out of season and alert threshold was the level above which influenza activity was higher than most years [1]. Using Excel, the transmission peaks of 5 years of data were aligned around the median week of peak occurrence during the influenza season for each year studied. Then we calculated the average weekly influenza positivity rate for each week centered on the median peak week of transmission. A 4-week running average was used to smooth the curve that represented the average epidemic curve. The standard deviation (SD) of the mean for each week was calculated to define extreme values, after which a curve was created for those values based on 1.65 SD above the mean representing the upper 90% confidence interval of the mean. This value was used as the alert threshold for severe seasons. After this procedure, weekly data for 2012 was plotted to the curve to determine how 2012 compared with the average epidemic curve, seasonal and alert thresholds calculated from the previous 5 years of observation.

### Comparison of circulating influenza viruses with Northern and Southern Hemisphere vaccine antigens

Comparison was done by simply matching the influenza viruses by type, subtype and/or lineage (in the case of influenza B) and by circulating strain for each surveillance year with the Northern (NH) and Southern Hemisphere (SH) vaccine antigens recommended for the influenza seasons for the same surveillance year.

## Results

### Number of ILI cases enrolled from 2006 to 2011

There were 8826 ILI cases enrolled in 2006. The numbers steadily increased through the course of surveillance and in 2011, 18,714 cases were enrolled. The highest number of ILI case enrolled was during the 2009 pandemic. The total number of ILI cases from 2006 to 2011 was 102,806. Mean specimen (NP or OP swabs) collection rate was 55.3% (Range 45.2 to 67.5%). Majority (91%) of patients with ILI who went to the sentinel sites were children under 15 years of age (Table 1).

**Table 1:** Number and age distribution of ILI cases from 2006 to 2011, National Influenza Surveillance, Philippines

Age group	2006 *N* (%)	2007 *N* (%)	2008 *N* (%)	2009 *N* (%)	2010 *N* (%)	2011 *N* (%)	Total *N* (%)
<6 months	624 (7.07)	944 (8.82)	2128 (9.74)	1732 (7.67)	1538 (7.64)	1623 (8.67)	8589 (8.35)
6–23 months	2525 (28.61)	3291 (30.74)	7156 (32.76)	6515 (28.86)	6064 (30.11)	5937 (31.72)	31488 (30.63)
2–4 years	2405 (27.25)	2846 (26.58)	5613 (25.70)	5511 (24.41)	5585 (27.73)	5390 (28.8)	27350 (26.6)
5–14 years	2393 (27.11)	2676 (24.99)	5175 (23.69)	6179 (27.37)	5258 (26.11)	4452 (23.79)	26133 (25.42)
15–49 years	757 (8.58)	746 (6.97)	1504 (6.89)	2269 (10.05)	1479 (7.34)	1110 (5.93)	7865 (7.65)
50+ years	89 (1.01)	113 (1.06)	246 (1.13)	313 (1.39)	198 (0.98)	200 (1.07)	1159 (1.13)
No data	33 (0.37)	91 (0.85)	22 (0.10)	56 (0.25)	18 (0.09)	2 (0.01)	222 (0.22)
Total	8826	10707	21844	22575	20140	18714	102806

### Influenza seasonality and duration

**Fig. 2:**
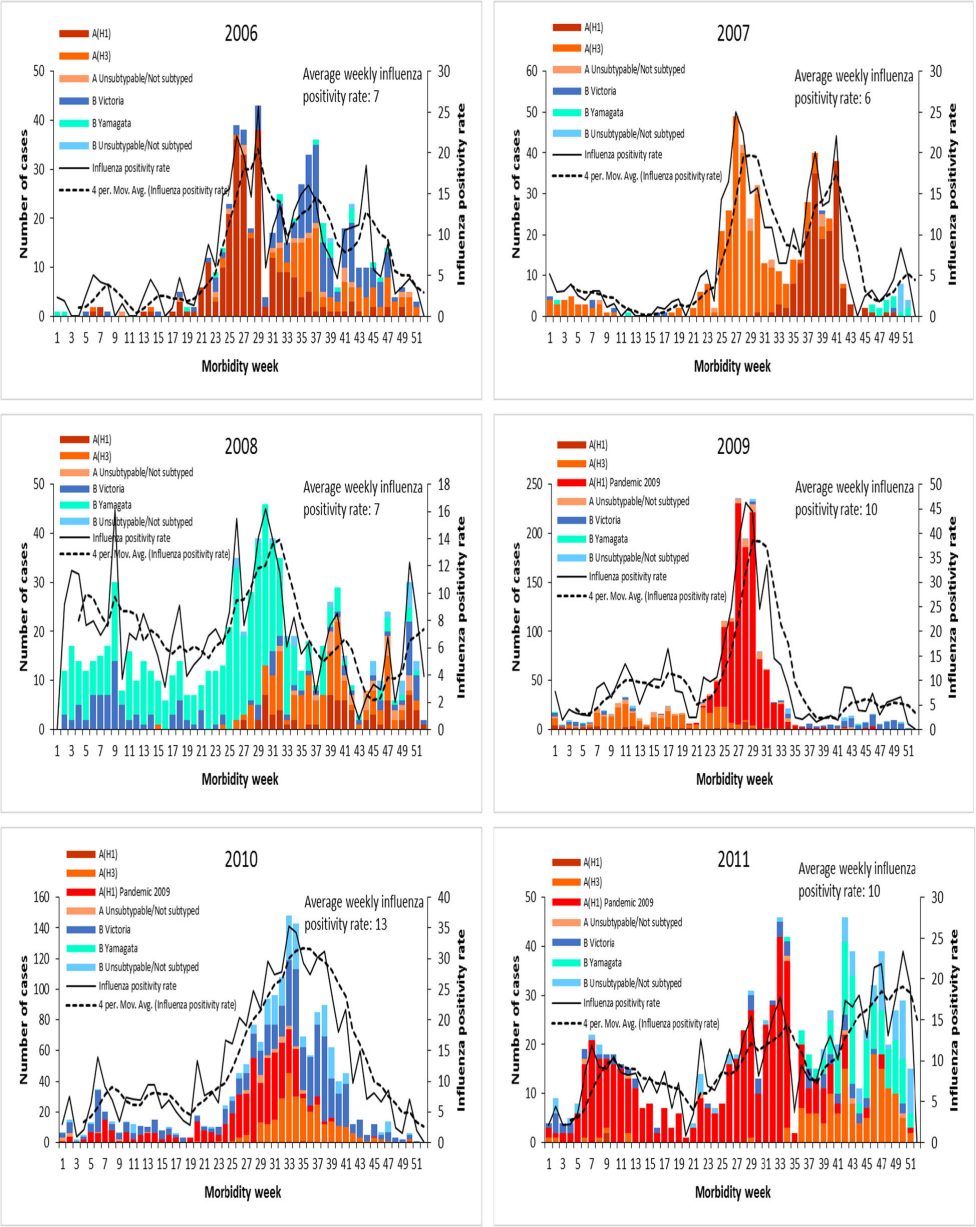
Plot of confirmed weekly influenza positivity rates from 2006 to 2011, National Influenza Surveillance, Philippines

Figure 2 shows the number of influenza virus positive ILI cases per week, weekly influenza positivity rates, 4-week moving average, average weekly influenza positivity rates for each year, and influenza types and subtypes for surveillance years 2006, 2007, 2008, 2009, 2010 and 2011. Excluding 2009, the pandemic year, the highest weekly influenza positivity rate was seen in 2010 (35%). Influenza virus activity was observed all-year round but with distinct weekly positivity rates above the average weekly influenza positivity rate for a year starting between weeks 24 to 31 and ending during weeks 41 to 52 (Table 2).

**Table 2:** Onset, end, duration and peak week of influenza season, 2006–2011. Influenza positivity rate by week and by month date

Year	Onset week (Month date)	End week (Month date)	Duration	Peak week (Month date)
2006	24 (Jun 11–17)	48 (Nov 26–Dec 2)	25	29 (Jul 16–Jul 22)
2007	25 (Jun 17–23)	43 (Oct 21–27)	19	27 (Jul 1–7)
2008	25 (Jun 15–21)	41 (Oct 5–11)	17	30 (Jul 20–26)
2010	24 (Jun 13–19)	44 (Oct 31–Nov 6)	21	33 (Aug 15–21)
2011	31 (Jul 31–Aug 6)	52 (Dec 25–31)	22	33 (Aug 14–20)
Median (week)	25	44	21	30
Range	24–31	41–52	17–25	27–33

Table 2 also shows the duration and peak weeks of the influenza season. The onset of the influenza season was not very different for the years 2006 to 2010. The end of influenza season however differed considerably between the years resulting in variation in the duration of influenza season. The year 2010 was an unusually severe season. This was also true for 2009 because this was the pandemic year. The median week of onset for the 5 years of surveillance, 2006 to 2011 (excluding 2009) was week 25 and the median week of the end of influenza season was week 44. The median duration of the influenza season was 21 weeks.

We observed that within the influenza season described above, there was more than one peak of increased proportion of influenza positive cases (Fig. 2). In 2006, using the 4-week moving average, three peaks were seen. The first peak was higher than the other peaks. Dual peaks were clearly distinct for 2007, 2008 and 2011. 2010 also had a minor second peak, which could only be detected by looking at the bar graph. Starting 2008 up to 2011, we observed minor peaks during the first 11 weeks of the year. However these were just single or two consecutive weeks of increased weekly influenza positive rates.

### Seasonal and alert threshold, and epidemic curve

**Fig. 3:**
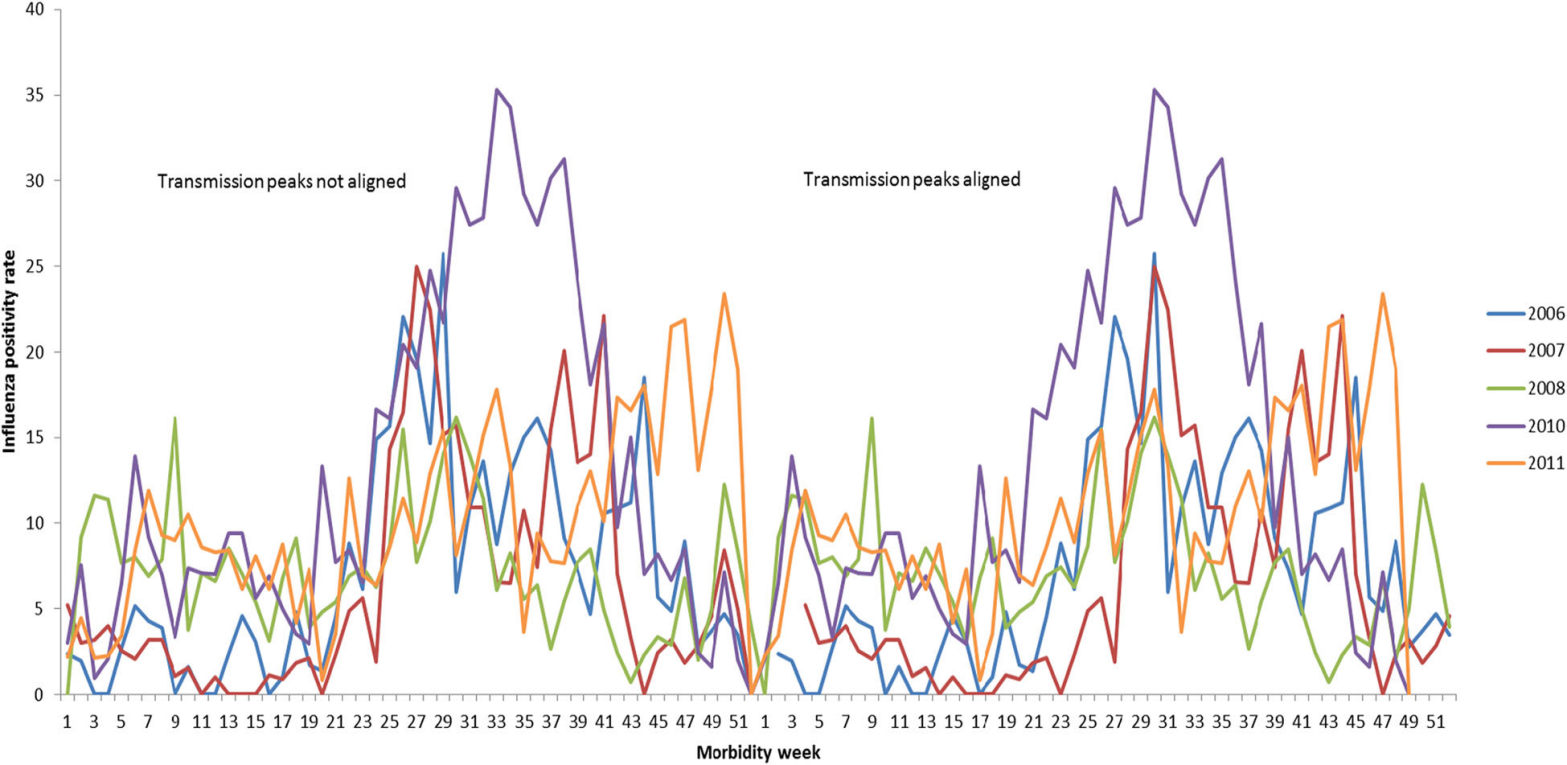
Weekly influenza positivity rates with transmission peaks of influenza activity aligned and not aligned

Figure 3 shows the non-aligned and aligned transmission peaks of influenza activity from 5 years of surveillance data.

**Fig. 4:**
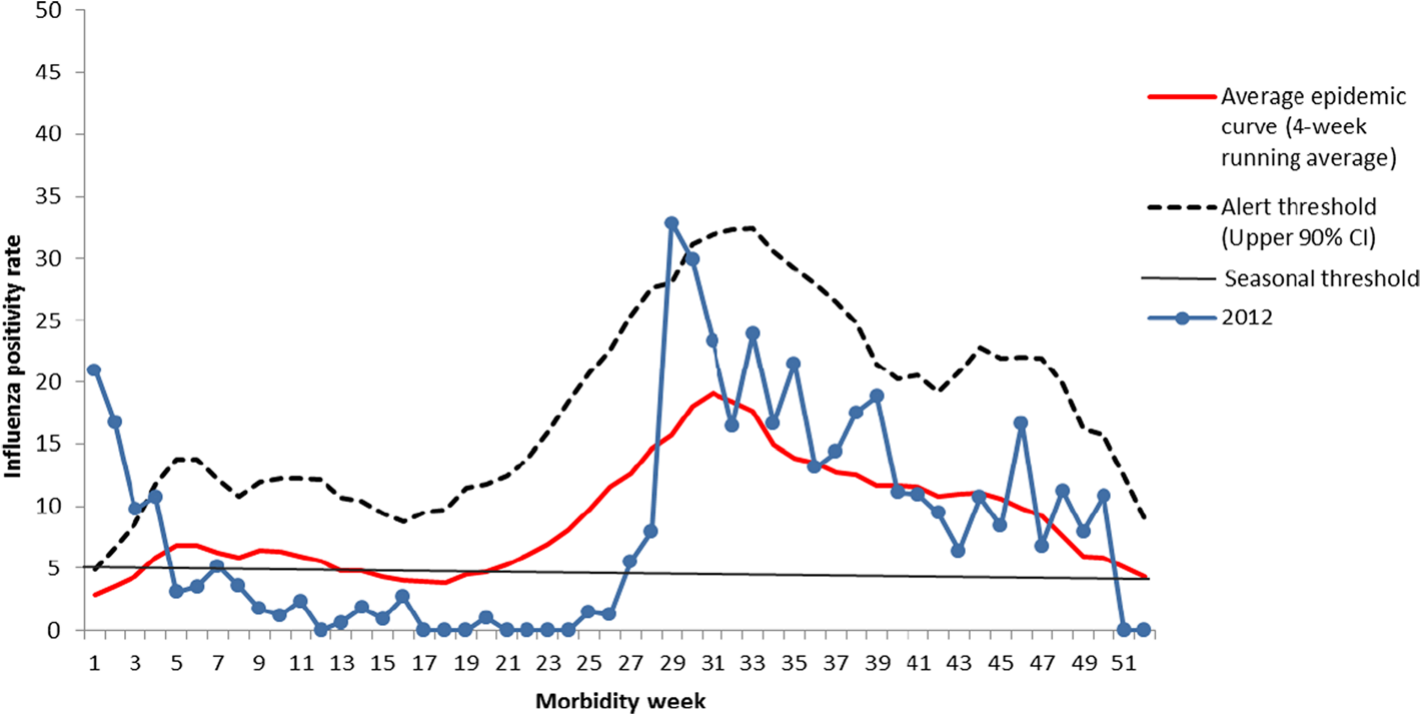
Plot of average epidemic curve, alert and seasonal thresholds and weekly influenza positivity rates for year 2012, National Influenza Surveillance, Philippines

Figure 4 shows the plot of percent of cases for 2012 compared to the seasonal threshold (5%), average epidemic curve and alert threshold. The first 2 weeks of 2012 showed weekly influenza positivity rates which were higher than the alert threshold followed by weekly influenza positivity rates that were lower than the seasonal threshold but only up to week 29 when influenza season started, 8 weeks after the expected week of rise of cases in week 21. The intensity was severe going above the average epidemic curve but of short duration. There was a sharp drop of weekly influenza positivity rates after week 30 lower than the alert threshold but still above the seasonal threshold. Thereafter, a minor peak was observed in week 35 conforming to the dual peak pattern observed for the other years. The decrease in the percentage of weekly influenza positivity rates below the seasonal threshold was sustained until week 40 with another small peak occurring in week 46.

**Fig. 5:**
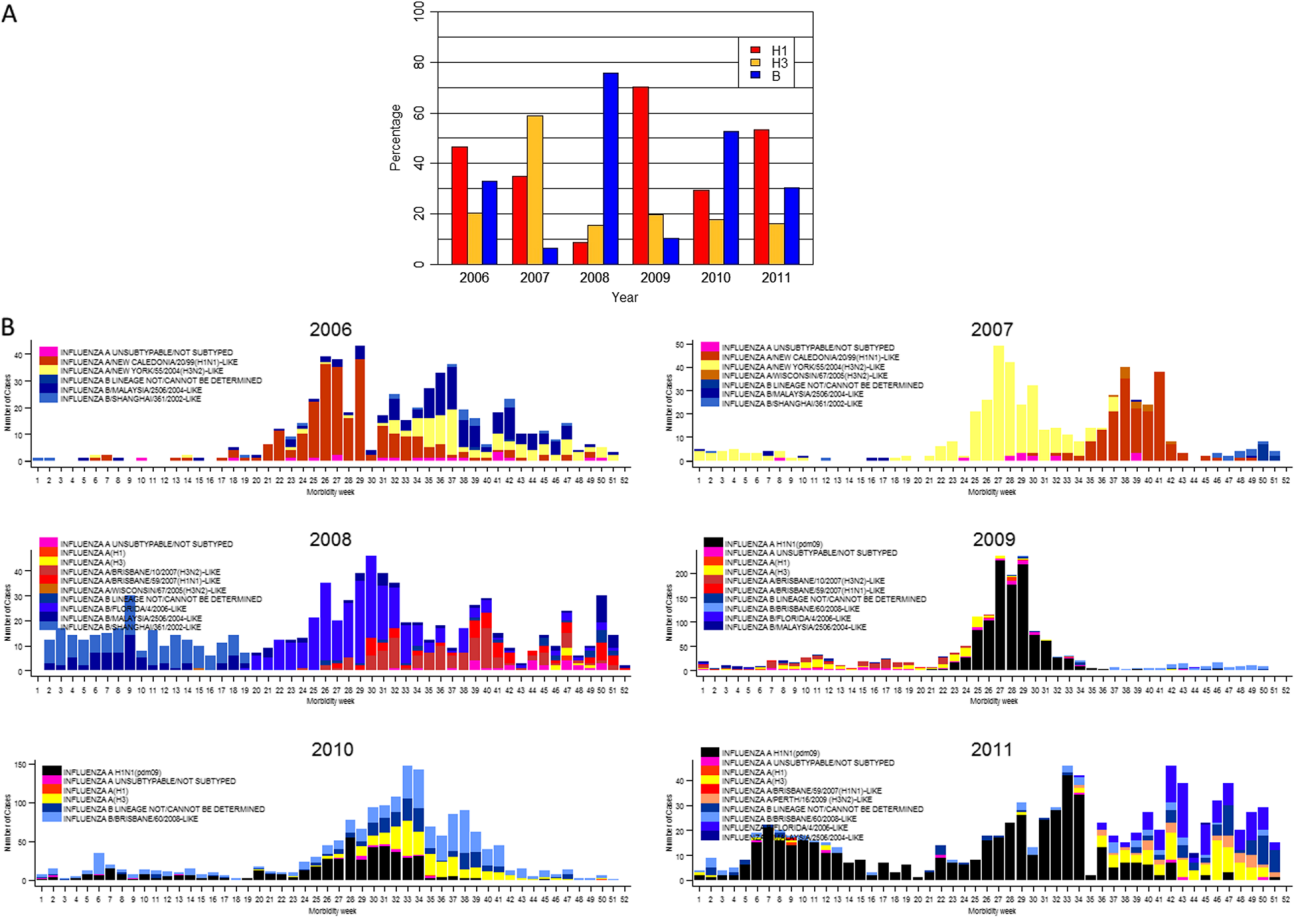
Panel **a**: Percentage of influenza subtypes from 2006 to 2011, National Influenza Surveillance, Philippines, Panel **b**: Circulating influenza strains from 2006 to 2011, National Influenza Surveillance, Philippines

Figure 5 Panel A shows that influenza A(H1) was the dominant subtype in 2006, 2009, and 2011, influenza B in 2008 and 2010, and A(H3) in 2007. In this panel, from 2009 to 2011, the red H1N1 bar includes both seasonal and pandemic influenza. Panel B shows that the periods or peaks of increased activity observed during an influenza season were each populated by distinctive influenza strains, either a single dominant or co-circulating strains. Two influenza B lineages were found for all years except 2009 although lineage of many influenza B strains could not be determined for this year.

### Comparison of circulating influenza strains and vaccine strains

Ninety-two percent of SH vaccine strains matched that of the circulating strains in 2006, compared to only 69% for NH strains. This was also observed in 2008 (69% versus 15.12%), and 2010 (66% versus 37%). Percent matched strains were lowest in 2009 (the pandemic year) with both SH and NH at 10.18% (Table 3).

**Table 3:** Comparison of Northern and Southern Hemisphere vaccine strains with the circulating strains per year of observation (2006–2011)

Northern Hemisphere vaccine strains	% Matching Circulating strains	Southern Hemisphere vaccine strains	% Matching Circulating strains
2005–2006		2006	
A/New Caledonia/20/99 (H1N1)-like	45	A/New Caledonia/20/99 (H1N1)-like	45
A/California/7/2004-(H3N2)-like ^a^	20	A/California/7/2004-(H3N2)-like^a^	20
B/Shanghai/361/2002-like virus (YAMAGATA)	4	B/Malaysia/2506/2004-like (VICTORIA)	27
Total Matching	69	Total Matching	92
^a^A/New York/55/2004(H3N2)-like virus was a circulating virus and was antigenically similar to A/California/7/2004-(H3N2)-like			
2006–2007		2007	
A/New Caledonia/20/99 (H1N1)-like	34	A/New Caledonia/20/99 (H1N1)-like	34
A/Wisconsin/67/2005 (H3N2)-like^a^	3	A/Wisconsin/67/2005 (H3N2)-like^a^	3
B/Malaysia/2506/2004-like	2	B/Malaysia/2506/2004-like	2
Total matching	39	Total matching	39
2007–2008		2008	
A/Solomon Islands/3/2006 (H1N1) like	0	A/Solomon Islands/3/2006 (H1N1) like	0
A/Wisconsin/67/2005 (H3N2)-like	0.12	A/Brisbane/10/2007 (H3N2)-like^a^	14
B/Malaysia/2506/2004-like (VICTORIA lineage)	15	B/Florida/4/2006-like (YAMAGATA)^b^	55
Total matching	15.12	Total matching	69
^a^A/Brisbane/59/2007(H1N1)-like virus (one of the H3N2 circulating strains) is not antigenically similar to A/Solomon Islands/3/2006 (H1N1)-like virus; ^b^B/Shanghai Yamagata lineage (circulating strain) is antigenically similar to B/Florida/4/2006-like virus			
2008–2009		2009	
A/Brisbane/59/2007 (H1N1)-like^a^	1	A/Brisbane/59/2007 (H1N1)-like^a^	1
A/Brisbane/10/2007 (H3N2)-like	9	A/Brisbane/10/2007 (H3N2)-like	9
B/Florida/4/2006-like	0.18	B/Florida/4/2006-like	0.18
Total Matching	10.18	Total Matching	10.18
^a^A(H1N1) pdm09 was the H1N1 circulating virus for 2009			
2009–2010		2010	
A/Brisbane/59/2007 (H1N1)-like	0	A/California/7/2009 (H1N1)-like^a^	29
A/Brisbane/10/2007 (H3N2)-like	0	A/Perth/16/2009 (H3N2)-like	0
B/Brisbane/60/2008-like virus	37	B/Brisbane/60/2008-like	37
Total Matching	37	Total Matching	66
^a^A/California/7/2009 (H1N1)-like is antigenically similar to A(H1N1) pdm09 which is a circulating virus			
2010 - 2011		2011	
A/California/7/2009 (H1N1)-like	53	A/California/7/2009 (H1N1)-like	53
A/Perth/16/2009 (H3N2)-like	4	A/Perth/16/2009 (H3N2)-like	4
B/Brisbane/60/2008-like^a^	6	B/Brisbane/60/2008-like	6
Total Matching	63	Total Matching	63
^a^B/Malaysia/2506/2004-like (Victoria lineage) another circulating strain is antigenically similar to B/Brisbane/60/2008-like			

## Discussion

In this study we have established the seasonal and alert thresholds, and average epidemic curve based on 5 years of influenza surveillance data using the method described in the WHO surveillance manual [1], described the seasonality of influenza in the Philippines, and presented the influenza strains circulating in the country from 2006 to 2011. While influenza viruses were detected year-round, increased activity was seen from June to November. We used the established thresholds and epidemic curve to assess the influenza season for 2012, its onset and end, and its severity in relation to these thresholds. We also showed that intensity of influenza activity was different for each year, and that different strains of influenza viruses circulated every year. Analysis of influenza activity using weekly detections showed more than one peak of activity within the influenza season from June to November of each year. Different dominant strains were associated with each peak. Two influenza B lineages circulated for five out of six surveillance years. Circulating strains matched the Southern Hemisphere vaccine more often than Northern Hemisphere vaccine strains.

The WHO surveillance manual has emphasized the need for standardized tools to establish thresholds and epidemic curves that could be used to compare influenza season between countries, and to assess the severity of the current season in a country to previous seasons of that same country. The method recommended in the manual [1] is relatively simple and uses software, which is readily available, and can be implemented by NICs as long as five or more years of influenza surveillance data are available. Knowing the usual baseline level of disease and the seasonal pattern as a point of reference aids in determining whether the current season is atypical both in timing and relative severity compared to previous ones. This information can help improve the accuracy of clinical diagnosis, appropriate use of antiviral medication, and the uptake and timeliness of seasonal influenza vaccines. As of this writing, we have not seen studies in Southeast Asia reporting established alert and seasonal thresholds or epidemic curves using the WHO surveillance manual. Although there is one publication from Thailand wherein Early Aberration Reporting System (EARS) and Cumulative sum (CUSUM) were used, calculations were only for ILI [22]. Routine influenza weekly monitoring reports from surveillance activities in Asia included ILI and influenza strains but compared these to historical influenza activity [23]. We have not been able to obtain information from countries in Asia on the use of the WHO method or moving epidemic method (MEM) to establish influenza seasonal thresholds or epidemic curves. At this point in time, there are already many countries in Asia, which have more than 5 years of surveillance data. The WHO method for calculating thresholds and epidemic curves could very well be applied in these countries.

The Philippines, Bangladesh, Cambodia, India, the Lao People’s Democratic Republic, Thailand and Vietnam have similar influenza seasonality [5]. Seasonality has been associated not only with climatological but also latitudinal variations [5, 24–26]. Influenza virus infection was found throughout the year in these countries, but more than 60% of influenza positivity rates were observed during the months of June to November [5]. The capital cities of these countries lie north of the equator (between 11.6 to 28.7° N). Thus, April to May is the most appropriate month for influenza vaccination for the Philippines and countries with similar seasonality [5]. In areas in India north of 30° N however, there was also increased influenza activity during winter, so that a separate vaccination timing has been recommended [5, 24, 25] for this country. In contrast, tropical countries in Asia below 11° N like Indonesia (Jakarta, 6.2° N), Malaysia (Kuala Lumpur, 3.2° N), and Singapore (1.3° N), influenza was found all throughout the year with no distinct influenza season [5]. Specific analysis on the association between influenza activity and climate variables has not been done in this study but June to November coincides with the rainy season in the Philippines similar to that of Cambodia [27], Myanmar [28], North Thailand [29], Bangladesh [5, 30], Lao [31], and some parts of India South of Srinagar [24, 25]. Factors other than the rainy season might also play a role for the increased influenza virus activity starting the month of June in the Philippines. Schools open in June for all levels in the Philippines, suggesting the effect of crowding on influenza transmission.

More than one peak of influenza activity was observed during the surveillance years but these peaks were confined to the defined influenza season from June to November. The presence of the different dominant influenza strains for each of the peaks may be through the following mechanisms: virus interaction leading to interference between viruses, break in transmission due to school vacation, development of immunity to the dominant virus, or a combination of these factors. Goldstein et al. [32] have noted that there was a negative association between strains’ incidences indicating that high infection rates with one strain can interfere with the transmission of other strains. Raoult [33] has postulated that the dynamics of influenza virus subtypes against those of other subtypes and even other respiratory viruses is complex and interference between these viruses might impact on their transmissibility in humans. School closure has been associated with reduced transmission of influenza virus because of decreased contact rate [34, 35]. School vacation or semestral break occurs at around the end of October to the first week of November. We have observed that influenza activity decreases during this time of school disruption. A break in the transmission of the first peak dominant virus causes co-circulating influenza strains to increase in activity leading to the development of a second peak. Increased transmission of the first dominant influenza strain may later lead to development of sufficient immunity among the population, which in turn will stop or decrease the spread of the virus. Consequently increased activity of co-circulating strains [36, 37] occurs.

The influenza strains circulating in the Philippines from 2006 to 2011 frequently matched the Southern rather than the Northern Hemisphere vaccine strains. The vaccine currently used in the Philippines is the Southern Hemisphere vaccine. Influenza B strains both of the Yamagata and the Victoria lineages were found during the years of surveillance. With these findings, the appropriate timing of vaccination should be from April to May, the current choice of Southern Hemisphere influenza vaccine should be continued, and most importantly, the vaccine should contain two influenza B lineages. Therefore, quadrivalent rather than trivalent vaccines would be a better choice.

There are several limitations to this study. The first limitation is that we used collated data from all sentinel sites to analyze seasonality. Sixty-four percent of influenza positive cases came from Luzon where Manila is located, 15% from the Visayas islands, and 21% from Mindanao. Since majority of the cases came from the Luzon islands, the results may be representative for only the north of the Philippines. The actual latitudinal location of the country extends from 5 to 20° N. Mindanao islands are situated below 9.7° N up to 5° N. The second limitation is that we were not able to compare the WHO method to other analytic methods. The third limitation is that data from this study mostly came from the pediatric age group (<15 years of age). There were very few adult ILI consultations probably because adults are able to handle ILI much better than children. The fourth limitation is that we could only match vaccine and circulating strains through names of the strains detected and not by genomic sequencing or antigenic testing. Although the Philippine National Surveillance followed the CDC definition for ILI, the definition of ILI was different from other countries and this may be considered a fifth limitation. Despite these limitations, this study has given a valuable insight into the measurement of seasonal and alert thresholds, and epidemic curves that will help DOH influenza program managers in the management of annual influenza epidemics in the country.

## Conclusions

Analysis of surveillance data from 2006 to 2012 has provided information about seasonal thresholds, epidemic curves, and circulating strains to guide health personnel in the control of influenza virus infection. Although influenza virus circulated throughout the year, a distinct seasonality was observed from June to November. The ideal time therefore to administer Southern Hemisphere influenza vaccines is from April to May. Quadrivalent vaccine might have more impact on influenza control than trivalent vaccine. Policy-makers are now capable of assessing the intensity or severity of the current influenza epidemic even early in its course, and plan more precisely resources necessary to control the outbreak. Influenza surveillance activities should be continued in the Philippines and funding for such activities should already be incorporated into the Philippine health budget. The results of seasonality patterns will be of immense help in the analysis of future studies on excess mortality during influenza seasons in the Philippines. Continuation of surveillance activities is needed not only to obtain data on circulating strains but also to recalculate the thresholds and epidemic curves as influenza surveillance continues. More sentinel sites should be added in the Mindanao islands. If possible, a separate analysis for seasonality is conducted for the Mindanao area. Further research should be conducted on the association of meteorological and other factors on seasonality of influenza virus infection. Genomic sequencing and antigenic testing of Philippine influenza virus isolates should be conducted to determine more accurately matching of vaccine and circulating strains.

## Additional file

Additional file 1: Data on influenza positivity rates from 2006 to 2011, National Influenza Surveillance Philippines and computation of average epidemic curve and alert threshold. (XLSX 52 kb)

## Abbreviations

BOD: Burden of disease
CDC: Centers for Disease Control and Prevention
CRF: Case report form
CUSUM: Cumulative sum
DMU: Data management unit
DOH: Department of Health
EARS: Early aberration reporting system
HAI: Hemagglutination inhibition
HSV: Herpes simplex virus
ILI: Influenza-like-illness
ISO: Influenza surveillance officer
MEM: Moving epidemic method
NEC: National Epidemiology Center
NH: Northern hemisphere
NIC: National Influenza Center
NP: Nasopharyngeal
OP: Oropharyngeal
RITM: Research Institute for Tropical Medicine
RITM-IRB: Research Institute for Tropical Medicine-Institutional Review Board
RSV: Respiratory syncytial virus
RT-PCR: Real-time reverse-transcriptase polymerase chain reaction
SD: Standard deviation
SH: Southern hemisphere
UK: United Kingdom
US: United States
VTM: Virus transport medium
WHO: World Health Organization
WHO-CCRRI: WHO Collaborating Centre for Reference and Research on Influenza
WHO-GISN: WHO Global Influenza Surveillance Network
